# The enduring consequences of conflict-related sexual violence: a qualitative study of women survivors in northern Uganda

**DOI:** 10.1186/s13031-022-00448-y

**Published:** 2022-04-11

**Authors:** Mahlet A. Woldetsadik, Grace Acan, Okwir Isaac Odiya

**Affiliations:** 1 Pardee RAND Graduate School, 1776 Main St, Santa Monica, CA 90401 USA; 2 Women’s Advocacy Network, Gulu, Uganda; 3 Justice and Reconciliation Project, Gulu, Uganda

**Keywords:** Northern Uganda, War, Sexual violence, Conflict-related sexual violence, Survivors, Women, Health

## Abstract

**Background:**

One in three women in northern Uganda report having suffered from conflict-related sexual violence (CRSV), including forced marriage and rape. Research on the long-term effects of CRSV on the health and social well-being of survivors is scant, but crucial to informing policy and improving programs tailored to conflict-affected communities. Understanding women’s perceptions of and experiences with CRSV, especially related to the persistent health and social challenges they continue to face, is critical for developing effective and targeted interventions.

**Methods:**

We worked with a local, survivor-led organization to recruit participants purposively from three post-conflict districts in northern Uganda: Gulu, Lira, and Pader. Women who had experienced CRSV and who were 18 years of age or older were eligible to participate. We asked participants open-ended questions about their experience with CRSV, including how it continues to affect their health and social well-being, any impact it had on their relationships, and if they faced barriers to accessing services. We transcribed, translated, and uploaded interview responses to the qualitative data analysis software MAXQDA and analyzed data thematically using a modified approach to grounded theory.

**Results:**

We conducted 30 interviews between October 2016 and March 2017. All participants reported experiencing forced marriage, rape, or forced pregnancy. Over two-thirds of participants said they continued to face physical and psychological issues, including untreated sexually transmitted infections, anxiety, and depression. Almost half of the women faced challenges with maintaining links with family members, stigma related to their experiences during abduction that also extended to their children born in captivity, and difficulty with accessing and affording health care. Barriers to seeking care included fear of disclosure and being unable to find services. Women identified peer-support from other survivors as a key coping mechanism.

**Conclusions:**

Women survivors continue to face multifaceted health and social problems in the post-conflict period. Most health-related programs that were set up at the end of the war in northern Uganda are no longer available. Increasing access to care, particularly services tailored to treating chronic reproductive health issues and mental health, is paramount for women survivors in northern Uganda and other conflict-affected regions.

## Background

Between 1986 and 2006, the Lord’s Resistance Army (LRA) committed large-scale human rights abuses in Uganda including killings, mutilations, abduction of children, and systematic sexual violence [1]. The conflict was mainly concentrated in the northern region and was a result of a widespread uprising against the National Resistant Movement (NRM), which was led by President Museveni who took office in 1989 after ousting Idi Amin [2]. Before the emergence of the LRA that was led by Joseph Kony, Alice Lakwena’s Holy Spirit Movement was popular and had more support than its successor. After Lakwena escaped to a refugee camp in Kenya, the LRA gained strength and became the most prominent rebel group against the government [2]. Conflict-related sexual violence (CRSV), defined as “rape, sexual slavery, forced prostitution, forced pregnancy, enforced sterilization, and other forms of sexual violence of comparable gravity perpetrated against women, men, girls, or boys that is linked directly or indirectly to a conflict” [3], was widespread during the 20-year conflict [4]. Although men and boys can also be victims of CRSV, women and girls are disproportionately targeted and constitute the majority of all survivors [5].

Over one-third of female youth (aged 14–35) surveyed in northern Uganda said they were abducted by the LRA [6] and 65% of formerly abducted girls reported experiencing sexual violence while in rebel captivity [7]. During the protracted conflict, most abducted females were forced to become “wives” to LRA fighters and commanders, experienced sexual abuse in the context of these unions, and had children as a result [7]. These experiences had detrimental effects on survivors including reproductive health problems, mental health issues, and social marginalization [4, 7, 8]. Studies have shown that women who were abducted during the conflict found it difficult to integrate back into their community due to stigma and were constantly trying to navigate strained family relations, which were disrupted by the conflict and subsequent displacement from their homes [9]. In addition, due to the absence of adequate health programming and psychological support and counselling, community rehabilitation has been a major challenge among conflict-affected communities in northern Uganda [9].

CRSV continues to be perpetrated in many conflicts around the world, with grave consequences. Between 1980 and 2009, there were 86 civil wars, where 18 of those conflicts involved “widespread rape” and 35 of those conflicts included “many or numerous reports of rape” [10]. For example, just in the past few years, extremist groups like Boko Haram in Nigeria, and the Islamic State of Iraq and the Levant (ISIS) in Iraq have abducted hundreds of girls and women who have suffered multiple forms of sexual violence in captivity [11–13]. Upon rescue, many of these girls and women were reunited with families who did not have the tools to cope with the trauma their loved ones experienced, as well as the adverse effects of the indirect exposure to sexual violence on themselves and other family members.

Experiencing CRSV can affect the social standing of survivors and families, expose them to stigma and discrimination, limit their access to social services, and prevent them from engaging in the labor market [14]. Moreover, families and communities often reject children born of rape in conflict, which might exacerbate survivors’ trauma and efforts to safely reintegrate into their former lives and communities [15]. Due to the risks, threats, and trauma associated with reporting CRSV, it continues to be dramatically underreported, especially in resourced-limited settings. Although there is some research on the health, social, and economic consequences of CRSV in sub-Saharan Africa [16, 17], there is still a lot to be learned about what the long-term effects of experiencing CRSV have been on survivors, including how spousal and familial relationships have been affected as a result of this experience.

Our aim was to assess the persisting impact of CRSV on the health and social well-being of Ugandan survivors from their own perspectives. We conducted in-depth interviews with women survivors in northern Uganda to explore the health and social challenges women continue to face, how their experience affected relationships with family members, and the types of support survivors sought or found the most helpful. Our hope is that lessons learned from the experiences of survivors in northern Uganda could be used to inform policy and improve health and other programing for post-conflict communities in Uganda, as well as other countries affected by CRSV.

## Methods

We followed the COnsolidated criteria for REporting Qualitative research (COREQ) guidelines on the design and reporting of qualitative research [18]. From October 2016 to March 2017, we conducted in-depth interviews with women survivors who lived in three post-conflict districts in northern Uganda: Pader, Lira, and Gulu. We asked participants open-ended questions about their experience with CRSV, including how it continues to affect their physical, psychological, and social well-being, and how it impacted their relationship with different family members. We also asked participants about the kind of support and services they sought or found the most helpful and suggestions they had for improving services tailored to survivors. We purposively recruited participants by working with leaders from the Women’s Advocacy Network (WAN), a survivor-led grassroots organization that works with and serves conflict-affected communities in northern Uganda. Women who had experienced at least one form of CRSV and who were 18 years of age or older were eligible to participate. We recruited participants until data saturation was reached, which was when no additional themes emerged after three consecutive interviews [19].

Before recruitment started, we conducted a small pilot to assess the practicality of proposed recruitment approaches, identify logistical problems, and field test the interview guides. The first author had previous experience working with communities affected by sexual violence in sub-Saharan Africa, including in post-conflict settings, and had extensive training in qualitative methods and field work. The second author and research assistant were both from northern Uganda, had experience working with conflict-affected groups in the region and conducting interviews with survivors of gender-based violence. The Ugandan research team was fluent in English and different dialects spoken in northern Uganda. Two interviews were conducted in English while the rest were conducted in either Acholi or Lango. Participants received transportation reimbursement of 10,000 Ugandan Shillings ($3), which was appropriate for this research in Gulu and surrounding areas. The study was approved by the Human Subjects Protection Committee at the RAND Corporation, the Institutional Review Board at Makerere University School of Public Health, and the Uganda National Council for Science and Technology in Oct 2016 (ref: SS36ES). We emphasized to participants that their participation was fully voluntary, and that we would respect their right to refuse to participate or discontinue participating once the interview started. We informed participants that their refusal to participate would not affect any services or support they were receiving from the Justice Reconciliation Project (JRP) or WAN. All participants gave oral informed consent to participate and have their interview audio recorded. Oral consent was preferred due to the sensitive nature of the issue being studied and potential risk a paper record could pose to participants. We also informed participants that psychologists from Gulu Women’s Economic Development and Globalization (GWED-G) were available to provide psychosocial care as needed.

Interviews lasted between 60 and 90 minutes and were digitally recorded, transcribed verbatim, and translated to English by a Ugandan translator who had prior experience working on similar research projects in northern Uganda. We analyzed data thematically in MAXQDA Analytics Pro 12 [20] using a modified approach to grounded theory [21, 22]. We developed a deductive codebook based on the main topics covered in the interview protocol to help organize the data and facilitate initial coding of the transcripts. MAW and GA used the first iteration of the codebook to code five transcripts independently and revised the codebook as new inductive themes emerged from the data. We reviewed the coded transcripts to ensure consistent application of themes and used an iterative process to resolve any coding discrepancies. This process helped us establish inter-coder reliability. In addition, the research team debriefed regularly to discuss the results and emerging themes. We also conducted a focus group with five participants to go over the results and seek their feedback. Focus group participants agreed with the emerging themes and provided practical suggestions for stakeholders working with CRSV survivors, which we incorporated into our final recommendations. We organized the codes thematically and used participant quotes to emphasize or support emerging themes as perceived by participants. When presenting quotes, we used pseudonyms to maintain participants’ anonymity.

## Results

Thirty women completed in-depth interviews. All of the women were abducted by the LRA between 1989 and 2005. The length of abduction ranged from 2 weeks to 9 years, and average time for the sample was 4.7 years. Participants in this study returned from abduction between 1996 and 2007. At the time of this study, it had been an average of 14 years since participants had returned. All participants reported experiencing at least two forms of CRSV during their captivity. These included forced marriage (partnership), rape (both within the context of forced marriage and by multiple rebels or government forces at different times), and forced pregnancy. The average age for the sample was 37 (range 23–60). The age at which participants reported being abducted ranged from when they were eight years old to 36 years old. Twenty-six women (87%) returned with at least one child; seven of these women came back with two or more children. At the time of the interview, 21 participants (70%) were either married on in a partnership. Full details on the key demographic features of all participants, including current marital status, occupation, and education is shown in Table 1.

**Table 1:** Characteristics of women survivors of conflict-related sexual violence (CRSV), northern Uganda (Oct 2016–Mar 2017)

Name*	Age	Age at abduction	Length of abduction	Married or in a partnership	Current occupation	Education	Bore child (ren)
Adong	60	34	NA	No	Farmer	Primary	Yes
Ajok	31	13	8 years	Yes	Unemployed	Secondary	Yes
Apiyo	35	8	9 years	Yes	Farmer	No education	Yes
Aol	32	14	6 years	Yes	Unemployed	Primary	Yes
Lamunu	49	30	N/A	No	Unemployed	Secondary	Yes (stillborn)
Piloya	30	12	5 years	No	Unemployed	Primary	Yes
Lajara	31	14	NA	Yes	Tailor	Primary	Yes
Adokorach	27	8	1.5 years	No	Farmer	Secondary	Yes
Lawino	30	9	NA	Yes	Unemployed	Primary	Yes
Alal	39	25	NA	Yes	Farmer	No education	Yes
Akello	34	12	6 years	Yes	Tailor	Primary	Yes
Lamaro	43	23	7 months	Yes	Self employed	No education	No
Aparo	33	13	8 years	Yes	Self employed	Primary	Yes
Amony	26	15	4 months	Yes	Farmer	Primary	No
Arac	31	11	8 years	No	Self employed	Primary	Yes
Akwero	23	10	2.5 years	Yes	Farming	Primary	Yes
Alanyo	34	14	6 years	No	Tailoring	Primary	Yes
Awor	45	19	1.8 years	No	Unemployed	Primary	No
Ayot	49	36	4 years	No	Unemployed	Primary	Yes
Amito	38	15	2 weeks	Yes	Farmer	Primary	Yes
Akiyo	33	16	2 years	Yes	Farmer	Primary	Yes
Ayaa	33	12	9 years	Yes	Unemployed	Primary	Yes
Acen	28	14	2 months	Yes	Farmer	No education	Yes
Aber	26	12	10 months	Yes	Unemployed	Primary	No
Acola	33	13	10 years	Yes	Tailor	Primary	Yes
Alum	33	13	NA	Yes	Farmer	Primary	Yes
Labol	33	14	6 years	Yes	Unemployed	Primary	Yes
Laruni	33	13	8 years	Yes	Tailor	Primary	Yes
Lamwaka	NA	NA	NA	No	Unemployed	No education	Yes
Lagum	37	14	7 years	Yes	Unemployed	Primary	Yes

*All names are pseudonyms to maintain confidentiality. Not Available (NA) was used when participants didn’t remember details or asked to skip the question

### Persistent health challenges

#### Physical health

Over two-thirds of participants (n = 27) raised issues surrounding persistent physical health problems related to their abduction and CRSV experience. These included chronic chest pain and untreated sexually transmitted infections (STIs). A participant who was abducted when she was 14 and returned with four children described the persisting chest pain she experienced which remains untreated due to her inability to afford medication,

My chest does not allow me to do hard work so I would need assistance for it to be checked. Unfortunately, my current job requires me to sit for many hours, sometimes I get difficulty in breathing. I need treatment to heal. [Alanyo, 34, Gulu]

Participants also shared suffering from recurring vaginal infections and incontinence due to injuries sustained after repeated instances of rape. A participant who was abducted at the age of 13 and returned with two children after nine years described the gynecological problems she continued to face,

One of the problems is that there might be damage in my bladder as a result of the flesh that came out at birth… maybe my bladder has enlarged, I do not know. I went to the hospital still with support from JRP [the Justice and Reconciliation Project] and I was given medicine but I still experience it. It does not flow uncontrollably like the case with fistula but I cannot hold urine. I am not seeking any medical service currently. As for my chest, I know that it cannot get cured. When it starts to hurt, and I feel pain, if I have money I buy medicine, otherwise I do not go to the hospital. [Acola, 33, Gulu]

Similar to Acola, most participants didn’t seek health care due to a range of structural barriers including lack of access to the type of care they needed, as well as high costs of medication.

#### Emotional and psychological health

Participants (n = 28) also raised issues around their emotional and psychological health. It was deeply concerning to them that they continued to be plagued by resentment and psychological disorders 10 to 15 years after returning from abduction. The most frequent psychological disorders participants experienced were anxiety and depression, followed by nightmares, occasional suicidal ideation, and thoughts of self-harm. Women described that most of these experiences were tied to their inability to forget what happened, which were exacerbated by insults and stigma in their community and their inability to care for the child(ren) they came back with. One woman shared,

Sometimes I dream that I am killing a person or that gunshots are soaring but prayers have helped a bit. Today I dreamt that we were being shot at and we ran and left behind our clothes…imagine these things happened many years ago but today when I get angry or think a lot, I relive these experiences that are fresh in my memory, especially when I am insulted about my life in the bush. [Apiyo, 35, Lira]

Participants shared that they were disturbed by their inability to move on and believed having these unresolved feelings affected multiple areas of their lives including their relationship with families, intimate partners, and their children born in captivity.

### Impact on relationships

#### Stigma

Participants had varied experiences when it came to the ways in which family members, intimate partners, and community members treated them (and their children) upon their return. Almost two-thirds of women (n = 19) said they were rejected by some family members, which severed close alliances, and at times led to the breakup of families and relationships. In some cases, this was because families and others in the community had the impression that survivors had killed people during their time in the bush and might have turned into a rebel themselves. A participant said,

Fear started building based on the assumption that when you stay in the bush, you have killed. They asked me whether I had killed but I did not kill anyone. For sure I beat someone under orders and because if I didn’t I could have been beaten as well but I did not kill anyone, unless it happened during battle for which I cannot know. [Aparo, 33, Gulu]

These negative sentiments also extended to survivors’ intimate relationships where how others perceived them, including some family members, affected the relationship they had with their former spouses or new partners. Survivors’ past experiences made it hard for some family members to accept them (and their children) and in most cases, participants shared that what they experienced during abduction was used as a justification for their rejection. A woman shared,

People told my husband not to marry me because my mind was filled with Kony’s [LRA leader] activities. When someone is interested in marrying me, they persuade him to not consider me because I returned from the bush. [Amony, 26, Lira]

#### Effect on intimate relationships

None of the participants in this study indicated having a positive relationship with their current husband or partner. Most women who were married or in a partnership reported difficulties in their current intimate relationship, which they ascribed to their past CRSV experience. Participants shared that in most cases, although their current partners generally knew that they had either been forcibly married or sexually abused during their abduction, they rarely discussed it. However, partners or spouses seemed to use women’s past experiences against them during arguments or misunderstandings, which participants said made them resentful of both the partner and the relationship in general. One woman shared,

Rape ruins relationship between husband and wife because each time you have a misunderstanding reference is made to the rape incident…insults like ‘you were raped’, ‘you were wife of the rebels’. It makes life difficult. [Amito, 38, Lira]

A few women described leaving relationships because the emotional abuse had become unbearable. However, most women in the sample said they decided to stay in relationships they did not want for economic reasons. They described that because of the many health and social challenges they were facing, they knew it would be difficult to support themselves and their child(ren) if they ventured out on their own. One woman said,

I am with a man but I have no love for men. It may be due to the current status of my relationship but I feel that I could be happier alone. At least if he was supporting me… but now sometimes he gives me UGX 5,000 for the day and sometimes nothing at all. At least when I was doing tailoring I could fend for us but now I can’t, all the children are at home. [Aparo, 33, Gulu]

#### Children born in captivity

More than 80% of women (n = 25) interviewed came back with children. Participants identified two major challenges related to coming back from abduction with children born during their captivity: issues related to the children’s (non)belonging and inability to inherit land and lack of financial support for the children’s education. Some women entered their new partner’s or husband’s home with children born in captivity. The presence of the children in new intimate relationships was often a source of conflict. Even in instances where some of the women got remarried, their new husbands didn’t support the education of the children who were born from captivity. One participant shared,

When they [family members] talk about the need to care for children, reference is made to her as not a child of that family. As a result, nobody supports her. He [current partner] is paying for his children’s schooling, but not the one I returned with from the bush. [Piloya, 30, Pader]

The issues of belonging were tightly tied with other greater problems women faced, including their male children’s inability to inherit land, as rights to land and inheritance in northern Uganda are deeply tied to paternal lineage. For children who don’t know their fathers or don’t have access to their fathers’ families, inheriting land is almost impossible. A woman shared,

The relationship is good with me but not with my children because there is no place for my child. The child does not know his clan, he does not know where to go. I say the relationship is bad because land is wealth, but no family member has told me, ‘here is land for the child that you returned with’. [Lawino, 30, Gulu]

Despite the uncertainty surrounding land inheritance for male children (17 women in the study came back with male children), most women in the study were hopeful that their sons would still be able to have a good future as long as they were educated. However, this was not easily achievable since they didn’t have the financial resources to send these children to school. Another participant said,

The problem that I am facing is paying school fees because there is no one to support me…. I worry that when she passes to go to secondary level, who will support me with her education? I see no prospects for her to continue to secondary level because I have not yet seen where I am going to get money. [Arac, 31, Gulu]

Other women shared similar sentiments and lamented their inability to secure financial resources for their children’s education. Most women sought assistance from local organizations and other support groups but were still unable to get any aid. However, few participants said they had some family support, and in addition, decided to look for the families of their child(ren)’s father in the hopes that they would want to be connected with these children. One woman shared,

When I returned, I was carrying a baby and there was nothing I could do about it. My mother comforted me. She said ‘my child, I’m glad that you returned, not your abduction or this child you are carrying was in your wish. If God wills and you give birth, we shall care for the child together’. [Akiyo, 33, Pader]

### Care sought

All participants were asked about their perspectives and experience with seeking health related care and other support. Specifically, they were asked about their access to care for their persistent physical and emotional health problems.

#### Barriers to care

Most women (n = 28) in this study shared that apart from the care they received when they entered through reception centers upon their rescue from the LRA, they did not continue to seek or receive formal care from health or other social institutions. Overall, participants identified three main personal and structural barriers including anxiety and fear related to sharing about the sexual abuse they experienced, not knowing how and where to look for help, and being unable to afford or find the type of health care they needed. One participant shared she worried people would use the information she would share about her experience against her and use it as a reason to mistreat her,

I don’t like to share issues. When something is pressing me… I run to the church and I cry to God because some people when you share with them your issue they use it to insult you. What I have found easy for me is running to God with prayers. [Lawino, 30, Gulu]

Others described not seeking care because they didn’t know how. This was especially difficult for women who did not tell anyone about the sexual abuse they experienced, which made it even more difficult to ask for help. Participants found it easier to share what happened to them once they realized that other women had undergone similar experiences. One woman shared,

I had a lot of anxiety and fear. I had decided not to share it but I learnt that so many people had gone through similar abuse. [Lajara, 31, Pader]

Cost of accessible services was another barrier to seeking and accessing care. A participant shared,

I have not tried seeking any assistance because even when I try I do not get. I tried with WAN [Women’s Advocacy Network]. WAN does not give money but we save our money in a group and counsel one another. [Akello, 34, Gulu]

### Suggestions for improvement

All participants provided suggestions for improving formal and informal services including access to health care and other coping mechanisms or support systems they found to be most useful. In addition to access to services for treating chronic physical health issues, participants identified three things they said helped alleviate the trauma they experienced during abduction and made the reintegration process easier. These included forgiving their abusers (and themselves), seeking out counseling from both professional providers and each other, and joining support groups led by women survivors. One woman described how she was initially filled with anger and resentment but started to forgive herself after going to church and getting counseling and support from church elders and other women in her community,

I started to release the poison in my life gradually when I went to church and got to know God. I started imagining the future of my child, I started planning for him but before that I saw him as a complete accident in my life. I would only think about what his father did to me and I would get so irritated. When I got saved by God is when I let go of every bad thing that was done to me, I started to forgive myself; I forgave my child; I started feeling relieved and that made me to forget some of the experiences I went through in the past. [Lawino, 30, Gulu]

Other women also shared similar narratives regarding their journey to self-forgiveness and healing. Getting counseling from women in their communities not only allowed them to forgive themselves, but also forgive those who abused them. For some women, it was easier to forgive their perpetrators when they accepted that perpetrators themselves had very little choice on decisions that were made by their superiors, which could have been the case in some instances since some of the rebels were forcibly abducted and turned into soldiers by the LRA. One participant shared,

I advise them to endure because above all we have survived, and that is important. They should avoid anger even when insulted. Forgive. Our rights were abused but we must forgive because some of the people who did that to us also did it against their will, they were also abducted. Let us console one another. [Lamaro, 43, Lira]

Another area where most women said they wanted support was counseling for survivors entering new intimate relationships. Participants described that there were complex challenges related to entering those unions with children born during captivity. In some cases, survivors either left these children with their families (e.g. mothers) or abandoned them altogether. A participant said,

You should help mothers who have returned, especially those who have found partners. Most of those relationships are not working out. We enter these relationships, have children then we break up. I am a victim of this problem. [Laruni, 33, Gulu]

#### Finding strength in each other

For nearly all women in the study, one of the most important sources of support was found amongst other women who had also been abducted and shared similar experiences of abuse. Through organizations like WAN that have worked at the grassroots level to destigmatize attitudes towards survivors of CRSV, participants said they were able to connect with each other and find strength through sharing of experiences. Most participants had a hard time talking about their experiences with their family members and spouses for a variety of reasons including fear of judgment, being blamed for what happened to them, and a strong sense (of resentment) that people who did not experience similar abuse wouldn’t be able to truly empathize. However, they reported finding emotional support from other women. One woman described,

First of all, the love among us who have returned is great; we feel as though another person’s problem is our own. When a colleague is suffering it’s as if we are the ones suffering. That is why we are able to console and counsel each other with care. You leave feeling comforted. But if you take your issue to someone who did not go through a similar experience, the moment you leave is the time when the information you shared spreads out. [Akello, 34, Gulu]

## Discussion

The women in this study reported facing continued challenges related to their abduction and the different forms of sexual violence they experienced. A 2007 population-based survey conducted on attitudes about peace, justice, and social reconstruction in northern Uganda found health to be the priority for women affected by the conflict [2]. Unfortunately, many years later, women continue to experience health problems related to sexual and other forms of abuse they experienced. Most of the women in the study reported suffering from untreated chronic physical health problems including chronic chest and abdominal pain, untreated STIs, and other forms of physical disabilities associated with sexual abuse and physical assault. Similar findings were noted by Liebling-Kalifani et al. [23] who argued for the use of human right frameworks in order to address the dire reproductive and gynecological health needs of Ugandan women who experienced sexual violence as a direct result of the Ugandan conflict. Sexual abuse history is an important risk factor for later health problems, and women with a history of sexual abuse report a higher level of medical symptoms including pelvic pain, headaches, and a higher disability in all areas of functioning including work and home management [24]. Our results support findings that Ugandan survivors’ needs for specialized and continued gynecological and reproductive health care is still unmet [25].

In addition, most women in the study suffered from a range of sustained psychological problems directly triggered by their experience during abduction. This finding supports previous research that has shown high prevalence of psychological distress and mental disorders including post-traumatic stress disorder (PTSD), substance abuse, and suicidal ideation among survivors of sexual and gender-based violence living in areas affected by armed conflict [4, 26, 27]. It’s important to note that it’s difficult to discuss mental health care for Ugandans affected by conflict without addressing the larger context of mental health care in the country. Mental health services in Uganda are generally scarce and cannot meet the treatment need for 90% of Ugandans with mental illness [28]. Barriers to care in the country include lack of trained staff and effective treatments, as well as pervasive stigma towards seeking mental health care [29]. These barriers are amplified in the context of conflict, which makes it difficult for survivors to access care. Although there has not been ample evidence of effectiveness of mental health interventions for survivors of CRSV in low-income settings [30], survivor-led groups such as WAN have been successful with providing informal group therapy to CRSV survivors, creating awareness about mental illness associated with CRSV, and reducing stigma around seeking mental health care. In addition, a randomized controlled trial of cognitive processing therapy (CPT) among Congolese survivors of sexual violence was found to reduce PTSD, depression, and anxiety symptoms as well as improve functioning [31]. This study was one of the first to show psychotherapeutic treatments such as CPT could be successfully implemented in low-income, conflict-affected settings with few mental health professionals. Given the similarity in both experience of CRSV and context, survivors in Uganda might benefit from a similar intervention.

When it comes to the impact of CRSV on relationships, most participants reported facing stigma and poor relations with family members and intimate partners as a result of their experiences. Survivors identified a few factors that negatively affected relationships including disclosure of CRSV experience and misguided ideas people had about former abductees turning to rebels. These findings imply that women who were abducted by rebel forces may be more prone to facing compounded stigma due to people’s views towards former abductees. This is consistent with studies that have shown that for women who return from abduction, especially with children born in captivity, re-establishing relationships with family and communities is a difficult process [32]. In terms of intimate partnerships, most participants revealed that their relationships were challenging and were exacerbated when they entered these unions with children born in captivity. Some women shared they stayed in these relationships for mainly economic reasons and would have preferred to be alone if they could support themselves.

Moreover, stigma and severed relationships (specifically related to fear of disclosure) adversely affected the general care seeking behavior of women in this study. Similar observations have been made in northern Uganda, where poor functioning among formerly abducted girls was found to be largely mediated by stigma and poor community relations [7]. With regards to care seeking behavior, most participants noted that lack of trust was one of the main factors that dissuaded them from seeking care. In addition, lack of access to medical services and cost of continued care were identified as major barriers. In addition to stigma surrounding sexual violence and being a former abductee, other studies have also identified lack of means to access medical care and awareness of available services to be some of the main structural barriers to seeking care [33, 34].

It is promising, however, that women found strength and support from joining women’s groups led by other women who were survivors of CRSV. Being part of these groups not only helped them feel less alone in their experiences, but also enabled them to freely discuss the challenges they were facing in a safe and non-judgmental environment. In addition, the social support they found amongst each other helped them develop positive coping skills, which helped mitigate some of the negative effects of the trauma they experienced [35].

Our study had a few limitations. Data were collected from women survivors who were identified by WAN and had somehow successfully found support through WAN and similar groups. Thus, the perceptions and experiences reflected in this assessment might be biased toward those who were willing and able to participate in the interviews and their concern may underrepresent the magnitude and scope of barriers faced by CRSV survivors that might not access support due to stigma and other barriers. Therefore, these interviews might not have captured the experience of the most vulnerable of survivors. Finally, this study only focused on women survivors living in three districts that were impacted by the Ugandan conflict and specifically by the LRA, and might not be generalizable to other survivors where the LRA was not the main perpetrator as well as men survivors of CRSV. Further research with larger and more diverse sample is needed to fully examine if what was observed in this study is applicable to the larger population of CRSV survivors in northern Uganda.

## Implications for policy, research, and practice

The challenges faced by survivors and their families can only be addressed through the collaboration of multiple stakeholders working with conflict-affected communities. These include local governments, non-governmental organizations, faith leaders, traditional elders, care providers, community workers, researchers, as well as survivors and families themselves. With this in mind, and drawing on the findings from this study, we present a set of key recommendations that could feasibly be implemented by the aforementioned stakeholders.

1. *Integrate mental health care and services focused on treating chronic physical health problems associated with sexual abuse into general health care and existing community support mechanisms* Targeting survivors specifically for specialized care can make them susceptible to stigma and discrimination. Program funders and implementers should support developing services for CRSV survivors that are integrated with other health care and implemented based on participatory principles and with the feedback and support of communities.
2. *Invest in improving the capacity of community workers so they can provide effective psychosocial care to survivors in their communities* Given that leaders of self-started survivor-led groups are providing informal care to survivors, donors and program implementers should enhance the capacity of these leaders through training in case management and psychosocial support.
3. *Reduce the stigma towards survivors of sexual violence and children born of war by working with faith leaders, and traditional elders* Community leaders, including faith leaders and traditional elders, have influence in the community that allows them to dispel stigma and shame projected toward survivors of sexual violence and children born in captivity. Program implementers should work with these leaders and with survivors to establish successful stigma-reduction interventions.
4. *Consider implementing interventions that use evidence-based psychotherapy techniques that have proven effective in reducing PTSD, depression, and anxiety symptoms in similar settings* Group psychotherapy techniques have been proven effective and cost-efficient in low-income and conflict-affected settings. Organizations already working with communities in northern Uganda should consider using similar interventions among survivors and families there.
5. *Encourage community dialogues around how war, abductions, and sexual violence can alter relationships* Family members and friends of survivors can take better care of survivors if they know what to expect during and after caring for traumatized people. Risk-reduction strategies such as strong social connections and counseling can protect individuals who care for survivors from secondary trauma.
6. *Increase survivors’ access to economic opportunities* Funders and program implementers should consider ways to provide education and vocational training so that survivors can diversify their skills and obtain resources for self-sufficiency.
7. *Provide access to land rights for women survivors and children born of war* Activists and survivors should work with traditional elders and community leaders to challenge traditional land ownership customs and lead reconciliation efforts that can reunite children with their paternal families.
8. *Support survivor-led grassroots organizations* Organizations such as WAN have helped destigmatize what it means to be a CRSV survivor or a former abductee. They have also helped reunite children born of war with their paternal families. These promising efforts should be supported both financially and through enhanced resources and training.

## Conclusions

This study demonstrated that survivors of CRSV continue to face multifaceted problems in the post-conflict period. Participants faced challenges with maintaining strong links with family members and intimate partners, stigma related to their experiences during abduction that also extended to their children born in captivity, and difficulty with accessing and affording sustained health services. However, participants were able to get a strong sense of emotional and moral support from other women who had similar experiences. Most health-related programs that were set up at the end of the war in northern Uganda are no longer available and the current programmatic priority is on transitional justice programs. However, none of the participants interviewed identified justice as a priority. Increased access to health services, particularly services tailored to treating chronic reproductive health issues and mental health, is paramount for women survivors in northern Uganda and other conflict-affected regions.

## Data Availability

The data used in this study are not publicly available due to research ethics board restrictions but are available from the corresponding author on reasonable request and on attaining research ethics board amendments from the RAND Corporation, IRB at Makerere University School of Public Health, and the UNCST.

## Abbreviations

CRSV: Conflict-related sexual violence
LRA: Lord’s resistance army
JRP: Justice and reconciliation project
WAN: Women’s advocacy network
